# Whole-Exome Sequencing Reveals Rare Germline Mutations in Patients With Hemifacial Microsomia

**DOI:** 10.3389/fgene.2021.580761

**Published:** 2021-05-17

**Authors:** Xiaojun Chen, Fatao Liu, Zin Mar Aung, Yan Zhang, Gang Chai

**Affiliations:** ^1^Department of Plastic and Reconstructive Surgery, Shanghai Ninth People’s Hospital, Shanghai Jiao Tong University School of Medicine, Shanghai, China; ^2^Bio-X Institute, Shanghai Jiao Tong University, Shanghai, China

**Keywords:** hemifacial microsomia, whole-exome sequencing, rare germline mutations, pathway enrichment analysis, mandibular hypoplasia

## Abstract

Hemifacial microsomia (HFM) is a rare congenital disease characterized by a spectrum of craniomaxillofacial malformations, including unilateral hypoplasia of the mandible and surrounding structures. Genetic predisposition for HFM is evident but the causative genes have not been fully understood. Thus, in the present study, we used whole-exome sequencing to screen 52 patients with HFM for rare germline mutations. We revealed 3,341 rare germline mutations in this patient cohort, including those in 13 genes previously shown to be associated with HFM. Among these HFM-related genes, *NID2* was most frequently mutated (in 3/52 patients). *PED4DIP*, which has not been previously associated with HFM, exhibited rare variants most frequently (in 7/52 patients). Pathway enrichment analysis of genes that were mutated in >2 patients predicted the “laminin interactions” pathway to be most significantly disrupted, predominantly by mutations in *ITGB4*, *NID2*, or *LAMA5*. In summary, this study is the first to identify rare germline mutations in HFM. The likely disruptions in the signaling pathways due to the mutations reported here may be considered potential causes of HFM.

## Introduction

Hemifacial microsomia (HFM), also known as craniofacial microsomia and oculo-auriculo-vertebral spectrum, is a rare congenital craniofacial malformation condition estimated to affect 1/3,000–1/5,000 live births (Birgfeld and Heike, 2019). Patients exhibit a broad spectrum of symptoms of varying severity, ranging from microtia to complex developmental defects of the face (e.g., maxillary and mandibular hypoplasia) and surrounding soft tissue. These can be further complicated by ipsilateral orbital anomalies, facial paralysis, transverse facial cleft, and other rare phenotypes (Tuin et al., 2015). HFM treatment is not only very painful and expensive but also challenging and predominantly symptomatic due to the complexity of induced defects.

The pathogenic mechanisms that underlie HFM remain unknown; however, HFM etiology is widely recognized as being multi-factorial, including both environmental and genetic factors (Beleza-Meireles et al., 2014). Despite the use of advanced gene sequencing technologies to identify chromosomal anomalies and candidate gene mutations in patients with HFM, the genetic causes of HFM remain elusive (Terhal et al., 2006; Kosaki et al., 2007; Zhu et al., 2007; Ala-Mello et al., 2008; Ou et al., 2008; Alasti and Van Camp, 2009; Rooryck et al., 2009; Huang et al., 2010; Northup et al., 2010; Rooryck et al., 2010; Su et al., 2012; Ballesta-Martinez et al., 2013; Quintero-Rivera and Martinez-Agosto, 2013; Torti et al., 2013; Zielinski et al., 2014; Beleza-Meireles et al., 2015; Colovati et al., 2015; Guida et al., 2015; Lopez et al., 2016; Zhang et al., 2016; Berenguer et al., 2017; Bragagnolo et al., 2018; Spineli-Silva et al., 2018). Thus, in the present study, we analyzed rare germline mutations by using the whole-exome sequencing (WES) technology to detect potential genetic causes and novel therapeutic targets for HFM.

## Materials and Methods

### Patients

**FIGURE 1:**
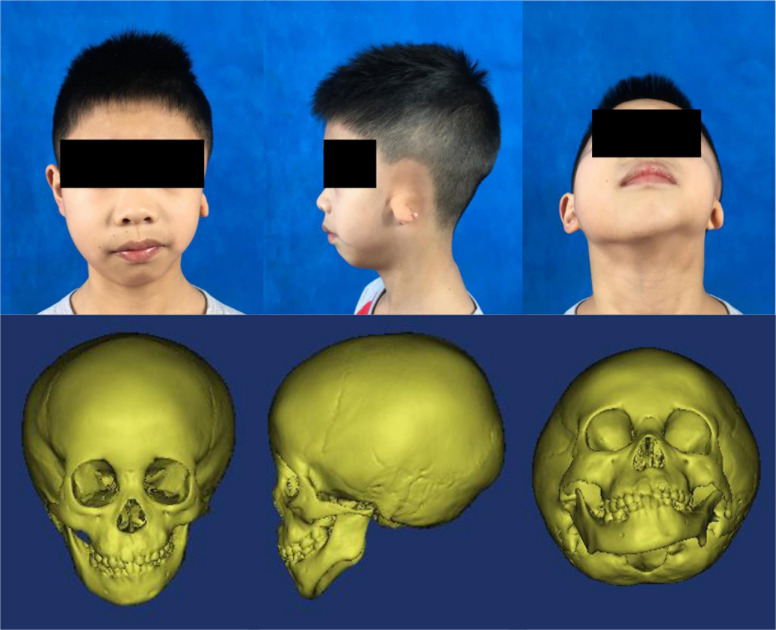
Typical photos and computed tomography scan images of a patient with hemifacial microsomia. From left to right: front, left-lateral, and upward view.

Although no common HFM diagnostic criteria exist, most patients with HFM exhibit underdevelopment of the mandible, maxilla, ear, orbit, soft tissue, and/or facial nerve. In this study, our minimal diagnostic criterion was the presence of unilateral mandibular hypoplasia diagnosed by using computed tomography scans and excluded patients with extracranial symptoms to minimize differences caused by the presentation of multiple phenotypes. The study thus enrolled a cohort of 52 patients with a mean age of 4.99 years (range, 0.5–20 years) (Figure 1), male:female ratio of 30:22, and a right:left-side-affected ratio of 26:26. All patients were further classified by using the OMENS+ classification system for HFM (Tuin et al., 2015; Table 1). All patients (or patient guardians) provided written informed consent prior to their participation in the study, which was approved by the Ethics Committee of Shanghai Ninth People’s Hospital, Shanghai Jiao Tong University School of Medicine.

**TABLE 1:** Clinical information for the 52 analyzed patients with hemifacial microsomia.

**Patient number**	**Sample name**	**Sex**	**Age (years)**	**Symptom laterality**	**OMENS+ classification**
1	B12815834	M	3	L	O1M1E2N3S1
2	B12816089	M	0.5	R	O2M3E3N0S3
3	WGC096878U	F	1	L	O1M3E3N1S2+C1
4	WGC096879U	F	7	L	O0M2bE0N0S2+C1
5	WGC096880U	F	6	R	O2M2aE2N2S1
6	WGC096882U	M	0.5	L	O0M2aE0N0S1
7	WGC098141U	M	3	R	O1M2bE3N3S1+C1
8	ly4454	M	10	R	O1M3E3N2S3
9	ly4453	M	1	L	O1M2aE2N3S1
10	ly4693	F	2	L	O1M1E0N0S1+C1
11	ly4691	F	7	R	O0M2aE3N0S2
12	ly4692	M	11	L	O1M2bE3N0S1
13	ly4808	F	2	R	O0M2bE0N0S1
14	ly4809	M	4	R	O0M2aE0N0S2+C1
15	ly5117	F	17	L	O0M2aE0N3S3
16	ly5116	M	8	R	O1M2aE0N0S1
17	ly5115	M	18	L	O1M2bE3N0S2
18	ly5133	F	20	L	O2M2aE0N0S1
19	ly5112	M	7	L	O3M2bE3N2S2
20	ly5113	M	7	R	O1M3E1N0S3
21	ly5119	M	13	R	O1M2bE3N1S2
22	ly5172	M	7	R	O1M2bE3N0S1
23	ly5170	M	2	R	O3M2bE3N2S1
24	ly5171	F	2	R	O3M2bE3N3S2
25	ly5176	M	1	R	O1M3E3N1S2+C1
26	ly5178	F	4	L	O0M1E0N0S1
27	ly5174	M	2	L	O1M1E3N0S1
28	ly5173	M	1	L	O0M3E3N3S2
29	ly5175	F	1	R	O0M2bE2N0S2+C1
30	ly5268	F	2	L	O1M2aE0N0S1
31	ly5265	M	1	L	O1M2bE0N0S1+C1
32	ly5664	M	11	R	O1M2bE0N0S2
33	ly5758	M	6	L	O3M2aE3N0S2
34	ly5759	F	4	R	O0M1E0N0S2+C1
35	ly5830	F	3	L	O3M2aE3N3S2
36	ly5839	F	0.5	L	O1M2bE1N0S2
37	ly5838	F	4	R	O2M2bE3N0S2
38	ly5877	M	6	L	O3M2bE3N2S3
39	ly5900	M	8	R	O2M2aE0N0S2
40	ly6059	F	8	R	O2M2bE3N0S2
41	ly6058	M	7	L	O2M2bE3N0S2
42	ly6060	F	1.5	L	O3M3E2N0S3+C1
43	ly6070	M	5	R	O0M2aE3N0S1
44	ly6075	M	0.5	R	O2M2bE3N0S1
45	ly6077	M	5	L	O1M2aE0N0S1
46	ly6385	M	1.5	R	O3M3E3N2S3+C1
47	ly6278	M	1	L	O1M2aE1N0S1+C1
48	ly6676	M	0.5	R	O3M3E0N0S2+C1
49	ly6688	F	3	L	O1M2aE0N0S2
50	ly6694	F	8	R	O0M2bE1N0S2+C1
51	ly6711	F	0.5	L	O1M3E3N2S2+C1
52	ly6756	F	2.5	R	O0M2bE3N0S2

*M, male; F, female; L, left side affected; R, right side affected.*

### WES

Whole-exome sequencing was conducted as previously described (Chen et al., 2018). Briefly, DNA was extracted from patient blood samples by using a Qiagen DNeasy Blood & Tissue Kit (QIAGEN, GmbH, Germany). RNaseA (QIAGEN, GmBH, Germany) was used to prevent RNA contamination. The purity and quality of the extracted DNA were determined by electrophoresis in a 1% agarose gel and by using a NanoDrop spectrophotometer (Thermo Fisher Scientific, Waltham, MA, United States) and a Qubit fluorometer (Thermo Fisher Scientific, Waltham, MA, United States). The purified genomic DNA was then sheared, and the samples (500 ng) were subjected to further purification, end repair, 3’-end adenylation, indexed pair-end-adaptor ligation, ligation-product purification, and polymerase chain reaction (PCR) amplification. A WES library was constructed by using a SeqCap EZ capture kit (Roche) for exome capture and subjected to further PCR amplification, purification, validation, normalization, and pooling. An Illumina HiSeq Series Analyzer was used for library sequencing.

### NGS (Next-Generation Sequencing) Data Processing

Next-generation sequencing data processing was conducted as previously described (Chen et al., 2018), with minor changes. Briefly, BWA software (Li and Durbin, 2009) was used to map the generated sequencing reads to the human genome (hg19). VARSCAN2 software (Koboldt et al., 2012) was used to detect germline mutations in the BAM files (minimum coverage, 20; minimum variant frequency, 0.08; *P*-value, 0.05). The fpfilter module of VARSCAN was used to identify false-positive variations. The resulting high-quality mutations were annotated by using SNPEFF (Cingolani et al., 2012) and VEP (McLaren et al., 2016) software.

### Mutation Classification and Pathway Analysis

Annotated mutations were selected for further analysis if they exhibited an alternative allele depth ≥ 10 and a population alternative-allele frequency < 0.0005 and were predicted to incur a ‘‘High’’ or ‘‘Moderate’’ severity impact. Small insertions and deletions (INDELs) and single nucleotide variants (SNVs) not predicted to be ‘‘benign’’ and ‘‘tolerated’’ by POLYPHEN2^1^ (Adzhubei et al., 2010), SIFT^2^ (Kumar et al., 2009), and CADD (cadd.gs.washington.edu) (Rentzsch et al., 2019) software tools, respectively, were used for following analysis as rare, potentially causative mutations.

Pathway analysis was performed by using ConsensusPathDB software^3^ (Kamburov et al., 2013) by entering the official names of the detected SNVs and INDELs and by using all known human genes included in the software database as controls. The Reactome database^4^ was used as a reference database to evaluate pathway enrichment.

## Results

### Novel Mutations in the Known HFM Genes

**FIGURE 2:**
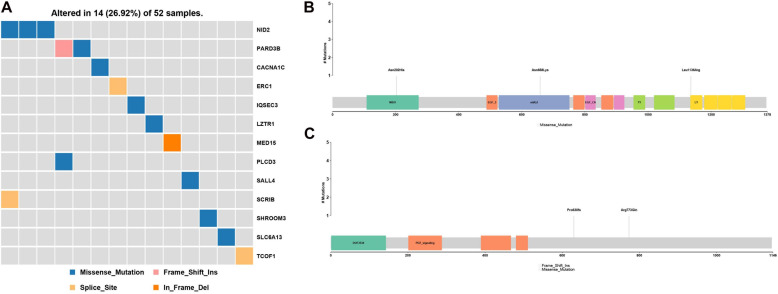
Gene mutations identified among the analyzed hemifacial microsomia (HFM) patient cohort. **(A)** A total of 13 known HFM genes were found to be mutated in the studied patient cohort, affecting 14 (16.92%) of the 52 analyzed patients. These included **(B)** three *NID2* mutations, Asn202His, Asn658Lys, and Leu1136Arg, and **(C)** two *PARD3B* mutations, Pro630fs and Arg773Gln.

PubMed database mining identified a number of gene and chromosome regions that have been previously associated with HFM (Table 2). Next, rare, potentially causative mutations that were detected in the 52 patients in the present study were screened to identify novel mutations in the known HFM genes. This analysis revealed 13 novel mutations in HFM-associated *NID2, PARD3B, CACNA1C, ERC1, IQSEC3, LZTR1, MED15, PLCD3, SALL4, SCR1B, SHROOM3, SLC6A13*, and *TCOF1*, which collectively affected 14 (26.92%) of the analyzed patients (Figure 2A). Among these, mutations in *NID2* affected the largest number of patients (3/52). The identified *NID2* mutations, Asn202His, Asn658Lys, and Leu1136Arg, were each located in different protein domains (Figure 2B). The PARD3B mutations Pro630fs and Arg773Gln were also detected in more than one patient (Figure 2C).

**TABLE 2:** Summary of previous genetic studies of hemifacial microsomia.

**Study**	**Mutated gene(s) symbol**	**Abnormal chromosomal region(s)/chromosome abnormality**	**Mutation type**	**Utilized analysis method**
Bragagnolo et al., 2018	*HMX-1, BAPX1, EYA1, PUF60, NRBP2, SCRIB, GATA3, ATR-16, NF1, PRKX, RAS2*	4p16.1, 4p16.3p15.33, Xp22.33–p22.31, 22q11.21, 8q13.3, 8q24.3, 10q26.2q26.3, 10p13p14, 16p13.3, 16p13.11p12.3, 17q11.2, Xp22.33	CNV (dup/del)	Karotyping with G-banding, CMA
Spineli-Silva et al., 2018	*YPEL1, MAPK1, ERK2, GSC2*	22q11.2	CNV (del)	MLPA, CMA
Berenguer et al., 2017	*MYT1*	20q13.33	SNV (c.323C>T, p.Ser108Leu)	SNP array
Lopez et al., 2016	*MYT1*	20q13.33	SNV (c.314C > T, p.Ser105Leu)	WES, transient knockdown in zebrafish
Colovati et al., 2015	*ZNF74, KLHL22, MED15, SNAP29, LZTR1*	22q11.21	CNV (del)	Genomic array analysis, MLPA
Zhang et al., 2016	*ROBO1, GATA3, EPAS1, PARD3B, GBX2, SHROOM3, FRMD4A, FGF3, KLF12, EDNRB, NID2, SEMA7A, PLCD3*	3p12.3, 10p14, 2p21, 2q33.3, 2q37.2, 4q21.1, 10p13, 11q13.3, 13q22.1, 13q22.3, 14q22.1, 15q24.1, 17q21.31	UK	SNP, GWAS, GRAIL, DEPICT, qPCR, WGS
Beleza-Meireles et al., 2015	Not determined	22q11	CNV (dup/del)	aCGH
Guida et al., 2015	*ATP13A3, XXYLT1*	3q29	CNV (dup)	SNP array
Zielinski et al., 2014	*OTX2*	14q22.3	CNV (dup)	WES, SNP array
Torti et al., 2013	Not determined	22q11.2	CNV (dup/del)	aCGH, FISH
Ballesta-Martinez et al., 2013	*OTX2*	14q23.1	CNV (dup)	Linkage analysis in families with autosomal dominant inheritance, aCGH
Quintero-Rivera and Martinez-Agosto, 2013	Not determined	22q11.1–q11.21	CNV (tetrasomy)	FISH, aCGH
Su et al., 2012	*TCOF1*	5q32–q33.1	UK	PCR, direct sequencing
Rooryck et al., 2010	*IQSEC3, SLC6A12, SLC6A13, JARID1A, CCDC77, B4GALNT3, NINJ2, WNK1, HSN2, RAD52, ERC1, FBXL14, WNT5B, ADIPOR2, CACNA2D4, LRTM2, DCP1B, CACNA1C, SPRY2*	12p13.33, 47, XXX, Yp–q11.221. Yq11.222–q12, t(9;18) (p23;q12,2), 13q13.1	CNV (del/dup/trisomy/translocation)	aCGH, QMF-PCR
Northup et al., 2010	Not determined	inv(14) (p11.2q22.3)	CNV (inversion)	FISH
Huang et al., 2010	*BIR1C, OCLN*	5q13.2	CNV (del)	Illumina HumanCNV370 Genotyping BeadChip, qPCR
Rooryck et al., 2009	*WNT5B, CACNA1C*	del(12) (pter/p13.33)	CNV (del)	aCGH, QMF-PCR
Alasti and Van Camp, 2009	*BAPX1, GSC, Hfm*	4p15.33, 14q32.13, 14q32	UK	Review
Ala-Mello et al., 2008	Not determined	5p15.3–pter, 21q22.3–qter, 21q22.11q22.12.	CNV (del/dup)	FISH, aCGH
Ou et al., 2008	*SIX1, SIX6, OTX2*	14q22.3–q23.3; 13q21.31-q21.32	CNV (del/dup)	Karotyping, FISH, aCGH
Kosaki et al., 2007	*SALL1*	16q12.1	SNV (c.1256T > A, p.L419X)	PCR, direct sequencing
Zhu et al., 2007	*ZIC3*	Xq26.3	UK	Analysis of *Zic3* null mice
Terhal et al., 2006	*SALL4* (exon 3)	20q13.2	UK	PCR, direct sequencing

*aCGH, array-based comparative genomic hybridization; CMA, chromosomal microarray analysis; CNV, copy number variation; del, deletion; DEPICT, data-driven expression-prioritized integration for complex traits; dup, duplication; FISH, fluorescence in-situ hybridization; GRAIL, gene relationships across implicated loci; GWAS, genome-wide association study; MLPA, multiplex ligation-dependent probe amplification; PCR, polymerase chain reaction; QMF-PCR, quantitative multiplex fluorescence-PCR; qPCR, quantitative PCR; SNP, single nucleotide polymorphism; SNV, single nucleotide variant; UK, unknown; WES, whole-exome sequencing; WGS, whole-genome sequencing. XXX means XXX syndrome, also known as Triple X syndrome, and trisomy X, which is a rare and genetic disease. It is characterized by the presence of an extra X chromosome in each cell of a female.*

### Genes With High-Frequency Rare, Potentially Causative Mutations in the HFM Patient Cohort

**FIGURE 3:**
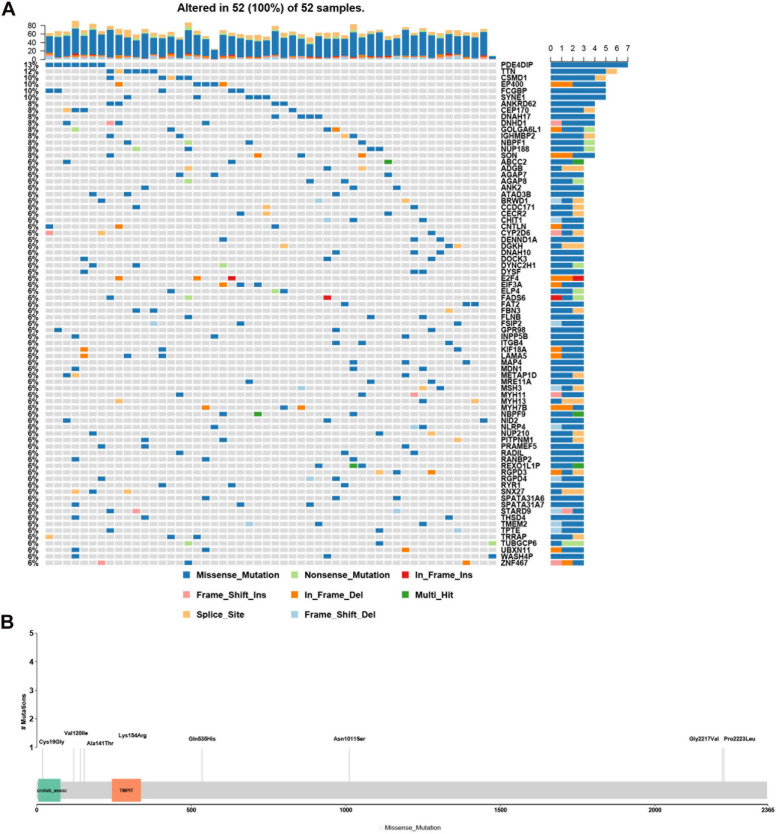
Analysis of rare, potentially causative mutations identified in the analyzed hemifacial microsomia (HFM) patient cohort. **(A)** List of genes found to harbor variants in >2 patients, ranked by mutation frequency. **(B)** Details of mutations (p.Ala141Thr, p.Lys154Arg, p.Cys19Gly, p.Asn1011Ser, p.Val120Ile, p.Gln535His, p.Pro2223Leu, and p.Gly2217Val.) in *PDE4DIP* that exhibited the greatest number of mutations and was the most frequently mutated gene among the analyzed patients.

Genes with rare, potentially causative mutations that were detected in more than two patients were ranked by mutation frequency (Figure 3A). Of these, the most frequently mutated gene was *PDE4DIP*. Its mutations p.Ala141Thr, p.Lys154Arg, p.Cys19Gly, p.Asn1011Ser, p.Val120Ile, p.Gln535His, p.Pro2223Leu, and p.Gly2217Val affected the largest number of patients (7/52) (Figure 3B).

### Pathway Analysis of Frequently Mutated Genes in the HFM Patient Cohort

**FIGURE 4:**
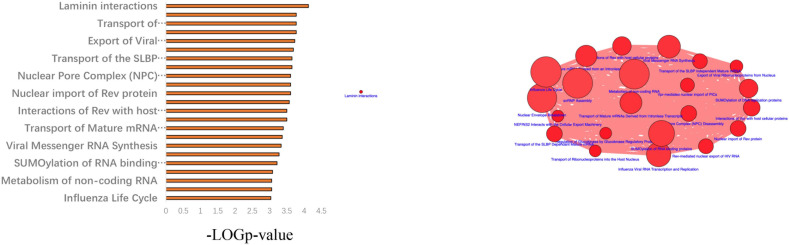
Results of the ConsensusPathDB enrichment analysis of genes found to be mutated in >2 patients with hemifacial microsomia (HFM).

**FIGURE 5:**
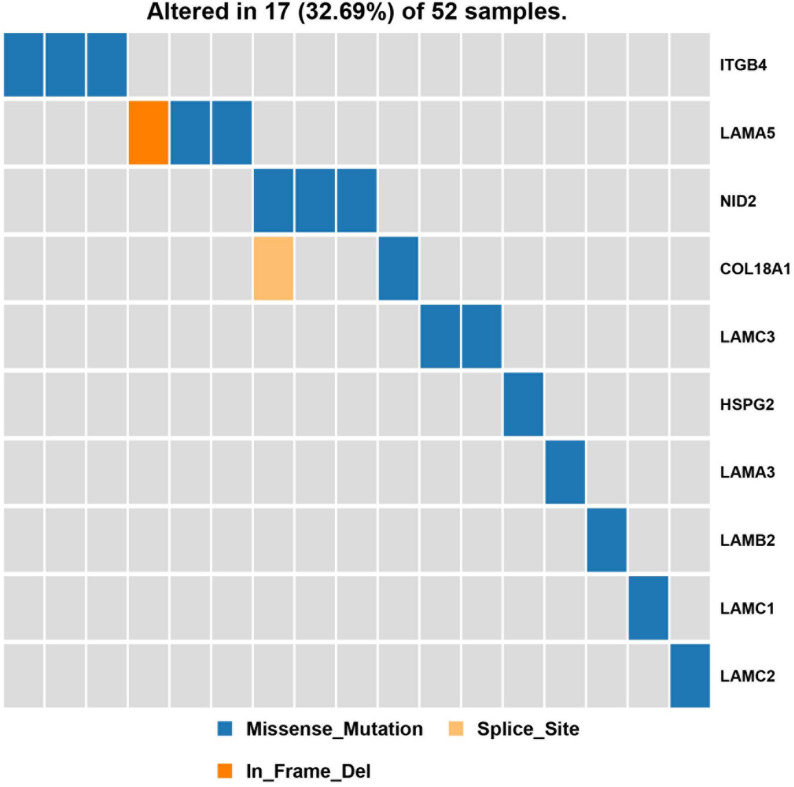
Results of the pathway enrichment analysis of genes found to be mutated in the analyzed hemifacial microsomia (HFM) patient cohort. The “laminin-interaction” pathway was predicted to be most significantly affected by the identified mutations (most often in *ITGB4*, *LAMA5*, or *NID2*) and was likely disrupted in 17 (32.69%) of the analyzed patients.

Next, a pathway analysis was conducted for all genes detected to harbor mutations among the 52 analyzed patients. This analysis showed that the most enriched pathways in the list of mutated genes were “extracellular-matrix organization,” “collagen-chain trimerization,” and “collagen formation” (Supplementary Figure 1). Genes mutated in >2 patients were also subjected to the ConsensusPathDB enrichment analysis (Figure 4), which found that the “laminin-interaction” pathway most frequently affected (17/52, 32.69%), predominantly by mutations in *ITGB4*, *LAMA5*, or *NID2* (Figure 5).

## Discussion

To date, the molecular mechanisms that underlie HFM pathogenesis remain unclear; however, strong evidence suggests that these include genetic factors, as evidenced by the fact that several chromosomal abnormalities and gene mutations have been previously reported in patients with HFM (Table 2). The largest study conducted to date was a genome-wide association study (GWAS) that was performed in 2016 (Zhang et al., 2016). However, that study encompassed all immature derivatives of the first and second pharyngeal arches. Thus, its results may not be closely related to the mandibular malformation. Furthermore, it is worth noting that very few risk variants identified by GWASs to date have been located in functionally significant protein regions (e.g., in the exons or the 5’-untranslated region) (McClellan and King, 2010). Moreover, the GWAS method is generally considered insufficiently sensitive to detect rare variants for rare diseases (McClellan and King, 2010). Given these shortcomings, NGS technologies such as WES have become increasingly important tools for studies aiming to elucidate the pathogenesis of rare diseases (Abul-Husn et al., 2016; Bilguvar et al., 2010; Bowden et al., 2010; Dewey et al., 2016; Lupo et al., 2019). Here, we have performed the first ever WES study of a large cohort of patients with HFM.

In the analyzed patient cohort comprising 52 individuals, we detected mutations in 13 genes that were previously associated with HFM, of which *NID2*, which encodes a member of the nidogen family of basement membrane proteins, was most frequently mutated. Previous studies have inferred a relationship between NID2 and osteogenic processes, consistent with the fact that the HFM phenotypic spectrum includes mandibular hypoplasia. For example, a previous study showed that *NID2* was markedly upregulated in demineralized osseous surfaces compared to its levels in mineralized osseous surfaces, suggesting that NID2 may act as a temporal migration guide (Wischmann et al., 2018). Furthermore, NID2 has been implicated in osteoclastogenesis. TRAP5α, which is involved in osteoclast signaling and RANK signaling in osteoclasts, has been shown to interact with NID2 in cultured 3T3-L1 mouse pre-adipocytes (Patlaka et al., 2014). The fact that patients harboring *NID2* mutations develop mandibular hypoplasia rather than mandibular agenesis is likely due to the fact that *NID1* and *NID2* are found in all vertebrates and exhibit partial functional redundancy. Consistent with this, genetic deletion of either gene alone induces only mild defects in the mouse (Bose et al., 2006).

Among the genes with rare, potentially causative mutations detected in the present study, those in *PDE4DIP* were found to affect the greatest number of patients in the analyzed cohort. Mutations in *PDE4DIP* have been previously identified predominantly during sequencing of tumor samples, e.g., in prostate (Gupta et al., 2017), ovarian (Er et al., 2016), or lung cancer (Li et al., 2015), as well as in adult pineoblastoma (Snuderl et al., 2018). To date, however, to the best of our knowledge, there have been no published reports implicating *PDE4DIP* mutations in HFM or bone formation.

Most previous studies investigating HFM pathogenesis have focused on a single gene mutation or mutations identified in a single patient; however, this approach is problematic given that the etiology of HFM is thought to be highly heterogeneous and dependent on genetic, epigenetic, and environmental factors. Thus, instead of focusing on single genes, in the present study, we conducted a pathway-based association analysis of generated WES data to identify common biological pathways that are likely to be affected in multiple (unrelated) patients with HFM. We suggest that this type of pathway analysis, which combines genomic and functional data and assesses the effect of multiple gene mutations, may be a better method to investigate the pathogenesis of rare diseases. Thus, we explored the distribution of signaling pathways known to be associated with genes that were mutated in >2 sporadic HFM cases. The “laminin-interaction” signaling pathway was predicted to be most frequently disrupted in the present cohort of patients with HFM, predominantly by mutations in *ITGB4*, *NID2*, or *LAMA5*. This is consistent with the fact that previous reports have demonstrated a close relationship (either positive or negative) between laminin interaction and bone formation during both osteogenesis and osteoclastogenesis (Langen et al., 2017; Susek et al., 2018).

The limitations of the present study are that candidate genes and pathways were not confirmed; further functional experiments are needed to confirm the candidate genes and pathways. Furthermore, it should be noted that the diverse phenotypic spectrum of HFM is thought to be the result of gene-environment interactions and etiologic heterogeneity, which collectively cause incomplete penetrance and variable expression (Birgfeld and Heike, 2019). Heterozygous mutations in reference alleles causing organ specificity in HFM may be affected by environmental and other factors. Many additional studies, such as RNA sequencing or whole-genome methylation studies, are needed to provide further information regarding this issue. In addition, we unfortunately failed to collect biological samples from the parents of the individuals involved. This was because the majority of patients came to our department with only one parent, and some of them refused to provide biological samples.

## Conclusion

In summary, this was the first study of rare germline mutations in a cohort of individuals with HFM. The likely disruptions in the signaling pathways due to the described mutations may be considered potential pathogenic causes of HFM and therefore, may serve as promising therapeutic targets.

## Data Availability Statement

The data presented in this study are deposited in NCBI online repositories. The BioProject ID is PRJNA626382. The SRA accession numbers for these isolates are from SAMN14639160 to 14639211.

## Ethics Statement

The studies involving human participants were reviewed and approved by Ethics Committee of Shanghai Ninth People’s Hospital, Shanghai Jiao Tong University School of Medicine. Written informed consent to participate in this study was provided by the participants’ legal guardian/next of kin. Written informed consent was obtained from the minor(s)’ legal guardian/next of kin for the publication of any potentially identifiable images or data included in this article.

## Author Contributions

XC, FL, YZ, and GC contributed to conception and design of the study. XC and ZM collected the clinical data and samples. FL performed the bioinformatics analysis. XC wrote the first draft of the manuscript. All authors contributed to manuscript revision, read, and approved the submitted version.

## Conflict of Interest

The authors declare that the research was conducted in the absence of any commercial or financial relationships that could be construed as a potential conflict of interest.

## Footnotes

1 http://genetics.bwh.harvard.edu/pph2/

2 http://sift.jcvi.org

3 http://consensuspathdb.org

4 https://reactome.org/

## Supplementary Material

Supplementary Table 1 Detailed information about all the mutations mentioned in the manuscript.
