# Supplementary figures and images for: Whole-Exome Sequencing Reveals Rare Germline Mutations in Patients With Hemifacial Microsomia
Source: Front Genet. 2021 May 17;12:580761. doi: 10.3389/fgene.2021.580761 (PMC8165440; doi:10.3389/fgene.2021.580761)

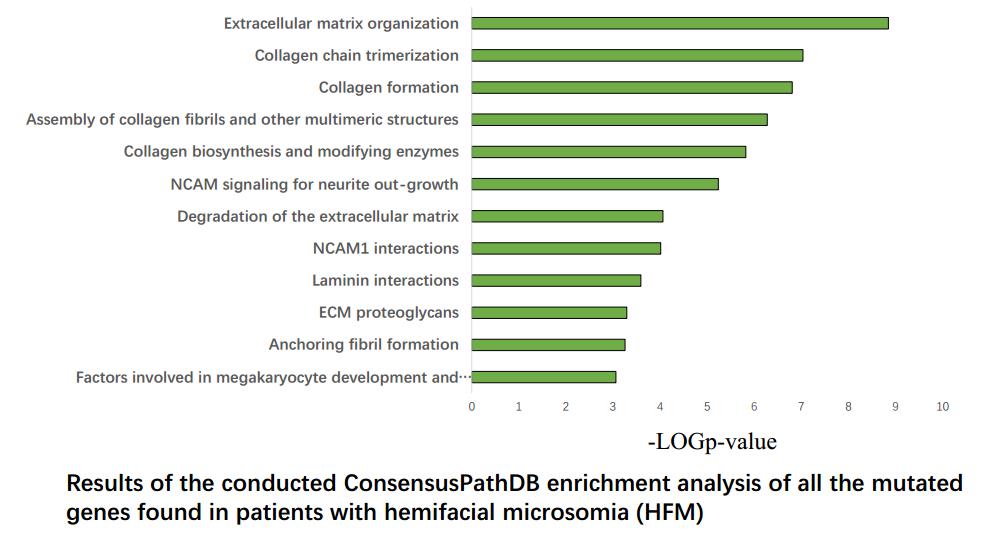

Supplement: Supplementary file 2 [file Image_1.JPEG]
